# The broad-spectrum chemokine inhibitor NR58-3.14.3 modulates macrophage-mediated inflammation in the diseased retina

**DOI:** 10.1186/s12974-016-0514-x

**Published:** 2016-02-24

**Authors:** Nilisha Fernando, Riccardo Natoli, Krisztina Valter, Jan Provis, Matt Rutar

**Affiliations:** The John Curtin School of Medical Research, The Australian National University, Building 131, Garran Rd, Canberra, ACT 2601 Australia; ANU Medical School, The Australian National University, Canberra, Australia

## Abstract

**Background:**

The activity of macrophages is implicated in the progression of retinal pathologies such as atrophic age-related macular degeneration (AMD), where they accumulate among the photoreceptor layer and subretinal space. This process is aided by the local expression of chemokines, which furnish these cells with directional cues that augment their migration to areas of retinal injury. While these qualities make chemokines a potential therapeutic target in curtailing damaging retinal inflammation, their wide variety and signalling redundancy pose challenges in broadly modulating their activity. Here, we examine the efficacy of the broad-spectrum chemokine inhibitor NR58-3.14.3—a suppressor of Ccl- and Cxcl- chemokine pathways—in suppressing macrophage activity and photoreceptor death, using a light-induced model of outer retinal atrophy and inflammation.

**Methods:**

Photo-oxidative damage was induced in SD rats via exposure to 1000 lux of light for 24 h, after which animals were euthanized at 0- or 7-day post-exposure time points. Prior to damage, NR58-3.14.3 was injected intravitreally. Retinas were harvested and evaluated for the effect of NR58-3.14.3 on subretinal macrophage accumulation and cytokine expression profile, as well as photoreceptor degeneration.

**Results:**

We report that intravitreal administration of NR58-3.14.3 reduces the accumulation of macrophages in the outer retina following exposure to light damage, at both 0- and 7-day post-exposure time points. Injection of NR58-3.14.3 also reduced the up-regulation of inflammatory markers including of Il6, Ccl3, and Ccl4 in infiltrating macrophages, which are promoters of their pathogenic activity in the retina. Finally, NR58-3.14.3-injected retinas displayed markedly reduced photoreceptor death following light damage, at both 0 and 7 days post-exposure.

**Conclusions:**

Our findings indicate that NR58-3.14.3 is effective in inhibiting subretinal macrophage accumulation in light-induced retinal degeneration and illustrate the potential of broad-spectrum chemokine inhibitors as novel therapeutic agents in thwarting retinal inflammation. Although broad-spectrum chemokine inhibitors may not be appropriate for all retinal inflammatory conditions, our results suggest that they may be beneficial for retinal dystrophies in which chemokine expression and subretinal macrophage accumulation are implicated, such as advanced AMD.

## Background

Age-related macular degeneration (AMD) is the leading cause of irreversible blindness among those aged over 65 in industrialised nations [1]. It is a progressive retinopathy that affects the photoreceptor population, retinal pigment epithelium (RPE), and Bruch’s membrane, and the choroid at various stages of the disease. The advanced stages of AMD are classified into two forms: exudative ‘wet’ AMD, induced by sudden choroidal neovascularisation (CNV), and geographic atrophy (GA) marked by a progressive expanding lesion of the RPE, photoreceptors, and choriocapillaris. While AMD has a complex aetiology involving multiple lifestyle and genetic risk factors, the significance of immunological processes has emerged slowly [2–7]. The relatively recent confirmation in genome-wide association studies (GWAS) of the importance of the complement system and macrophages has placed inflammation among the forefront of factors influencing both the onset and progression of AMD [8, 9].

The accumulation and activation of macrophages within the retinal tissues is a prominent feature of most retinopathies and is well documented in all forms of AMD. Microglial cells are the resident macrophages of the retina that are derived from embryonic yolk sac progenitors during development [10, 11], while non-resident bone marrow-derived macrophages may be recruited into the retina from the vasculature in pathology [12, 13]. In AMD lesions, macrophages form large aggregations in the photoreceptor layer, subretinal space, and RPE [4, 6, 7, 14–17], which are typically free of these leukocytes in healthy individuals [18]. Despite having some beneficial properties [19], excessive accumulation of these cells is implicated in degeneration in animal models of AMD, retinal detachment [20], diabetic retinopathy [21–23], and glaucoma [24, 25].

Chemokines are key regulators of macrophage activation and accumulation in AMD [26, 27], as well as degenerations associated with retinal detachment [20] and diabetic retinopathy [23]. The chemokine family comprises more than 50 molecules and associated receptors that activate and recruit leukocytes to sites of damage. Transcriptome-wide analysis of AMD donor retinas reports that the leukocyte chemoattractants Ccl2, Cxcl1, Cxcl10, and Cxcl11 are up-regulated in all forms of the disease [28]. Moreover, intraocular Ccl2 protein levels are increased in patients with CNV or GA [16, 29, 30], and infiltrating microglia/monocytes in retinas with GA express the cognate Ccl2 receptor, Ccr2 [16]. Chemokine signalling is implicated in exacerbating the accumulation of macrophages and photoreceptor death in a range of animal models that demonstrate some features in common with AMD, including laser-induced CNV [31], photo-oxidative stress [16, 32, 33], Abca4^−/−^Rdh8^−/−^ mice [34], and carboxyethylpyrrole (CEP)-immunised mice [35].

While these findings underscore the importance of chemokines in shaping inflammation in the retina, therapeutic blockade of individual chemokine ligands/receptors may have limited efficacy due to chemokine receptor redundancy, and the tendency toward compensatory increases in expression of other chemokine family members [34, 36]. Broad-spectrum chemokine inhibitors (BSCIs) are a class of chemokine modulators with the ability to inhibit signal transduction of a broad swathe of chemokine receptors [37]. Among these, NR58-3.14.3 is a robust cyclic peptide that specifically inhibits chemokine-mediated migration of macrophages by blocking the signalling of Ccl- and Cxcl- pathways [37]. NR58-3.14.3 has been shown to effectively inhibit macrophage accumulation and ameliorate pathology in lung ischemia [38], obliterative bronchiolitis [39], skin inflammation [37], and atherosclerotic plaques [40].

To our knowledge, there are no studies which have explored the potential of BSCIs in retinal degeneration models. Here, we investigate the effect of NR58-3.14.3 in modulation of the accumulation of macrophages in the outer retina, using a light-induced model of oxidative stress and retinal inflammation. While acute exposure to light damage does not induce some of the classical features of AMD (including drusen and CNV), it induces focal and progressive atrophy of the outer retina and models very effectively the accumulation of subretinal macrophages and up-regulation of chemokines Ccl2, Cxcl1, Cxcl10, and Cxcl11 [41]. Furthermore, we have shown that the degenerative changes associated with light damage closely mimic naturally occurring degenerative changes in the ageing rat retina [42].

In the current study, we show that intravitreal delivery of NR58-3.14.3 is safely tolerated in experimental animals, is long-lasting in the retinal environment, suppresses macrophage accumulation in the outer retina, and reduces retinal pathology following light damage. Additionally, we demonstrate that NR58-3.14.3 reduces the expression of pro-inflammatory factors Il6, Ccl3, and Ccl4 by macrophages, which are implicated in promoting their deleterious activity in retinal dystrophy. These data provide proof-of-principle support of the value of BSCIs in thwarting deleterious macrophage activity in retinal degeneration.

## Methods

### Animal handling and rearing

All experiments were in accordance with the ARVO Statement for Use of Animals in Ophthalmic and Vision Research and with approval from the ANU Animal Experimentation Ethics Committee (Ethics ID: A2014/56). Adult Sprague-Dawley (SD) rats were utilised in all experiments, which were born and reared under dim-light conditions (5 lux) and aged between 90 and 120 post-natal days at the time of use.

### Preparation of NR58-3.14.3 and intravitreal injections

Animals were anaesthetised with an intraperitoneal injection of ketamine (100 mg/kg; Troy Laboratories, NSW, Australia) and Xylazil (12 mg/kg; Troy Laboratories, NSW, Australia). Intravitreal injections of consisting of 3 μL were conducted as described previously [32]; this route was preferred over systemic administration as it minimises the potential for off-target effects which may cofound the data. The 3-uL injection volume was chosen as it offers good reproducibility, minimal disturbance in vitreal volume, and negligible cataract formation [43]. The broad-spectrum chemokine inhibitor (BSCI) NR58-3.14.3 (Auspep, VIC, Australia) was reconstituted in endotoxin-free 0.1 M PBS to a final concentration of 66.7 μg/μl. In the treatment group, 3 μl of NR58-3.14.3 in PBS was intravitreally injected into both eyes of each animal, so that 200 μg of NR58-3.14.3 was delivered to each eye. The control group received an injection of 3 μl PBS vehicle in each eye. Following intravitreal injections, the animals were monitored closely throughout the time course of experiments. As expected, no incidences of cataract formation or other abnormalities were observed.

### Light damage

Immediately after administering intravitreal injections, animals were exposed to 1000 lux light for a period of 24 h, as described previously [44]. During light damage, animals were placed into transparent Perspex open-top cages under a light source (COLDF2, 2 × 36 W, IHF, Thorn Lighting, Australia) with access to food and water ad libitum. After light damage, animals were either euthanized for tissue collection (0 day time point) or were placed into recovery under dim-light conditions (5 lux) for 7 days.

### Retinal localisation of injected NR58-3.14.3 via fluorescein

The fluorescein-labelled NR58-3.14.3 was prepared using an NHS-Fluorescein Antibody Labelling Kit (Pierce, Thermo Fisher Scientific, MA, USA), applied according to the manufacturer’s protocol. In brief, the NR58-3.14.3 was reconstituted in endotoxin-free PBS to a concentration of 1 mg/ml. The NR58-3.14.3 was then incubated with the NHS-Fluorescein for 1 h at room temperature, before being purified through a column to remove excess dye from the final product. The fluorescein-NR58-3.14.3 was stored at 4 °C. Using the intravitreal procedure described earlier, each animal was injected with 3 μl fluorescein-NR58-3.14.3 in one eye only; the contralateral eye was used as either a fluorescein-only-injected control, or a non-injected control. Following the injection, animals were placed under dim-light conditions to recover. Both eyes of each animal were collected for cryosectioning at several times after the injection (20 min, 3 h, 24 h, and 7 days). The levels of fluorescein-NR58-3.14.3 in injected retinas were measured on cryosections. The fluorescence was visualised with a laser-scanning A1^+^ confocal microscope (Nikon, Tokyo, Japan), and images were acquired at consistent regions using the NIS-Elements AR software (Nikon, Tokyo, Japan). From these images, fluorescence intensity was quantified with ImageJ software (NIH, MD, USA), using integrated density for a fixed area.

### Tissue collection and processing of whole retinas

Animals were euthanized using an overdose of barbiturate, which was administered via intraperitoneal injection (Valabarb; Virbac, NSW, Australia). The left eye from each animal was marked for orientation then enucleated for cryosectioning, while the retina from the right eye was excised through a corneal incision for RNA extraction. Both cryosectioning and RNA extraction of the excised retinas were performed according to our previous methodology [45].

### Fluorescence-activated cell sorting (FACS) of retinal macrophages

Rats in each treatment group were euthanized immediately after light damage (0 day) using barbiturate overdose as previously described. Retinas from both eyes were promptly removed through a corneal incision. Retinas from each animal were pooled and immediately placed in chilled Hank’s balanced salt solution (HBSS) and then subjected to light mechanical separation using a razor blade. Samples were transferred into a 0.2 % papain digestion cocktail as described in our previous protocol [41], with minor modifications. Samples were incubated at 37 °C for 7 min then 8 °C for 30 min. After neutralisation and resuspension, the resulting homogenate was incubated in staining buffer containing a CD11b antibody conjugated to Alexa 647 (Biolegend, San Diego, CA, USA) for 30 min at 4 °C, and then washed in HBSS and resuspended in staining buffer. The resultant CD11b-stained samples were run through a fluorescence-activated cell sorter (FACS) (BD FACSAria II; BD Biosciences, Franklin Lakes, NJ, USA). The isolated CD11b+ macrophages were collected in staining buffer and kept chilled on ice until RNA extraction could be commenced. RNA extraction was conducted via a protocol we have previously established [41]. Isolated total RNA was analysed for quantity and purity with a ND-1000 spectrophotometer (Nanodrop Technologies, Wilmington, DE, USA).

### TUNEL assay and quantification of photoreceptor survival

To quantify levels of photoreceptor cell death in the retina, retinal cryosections were stained for apoptosis using a terminal deoxynucleotidyl transferase dUTP nick end labelling (TUNEL) kit (Roche Diagnostics, NSW, Australia) as previously described [46, 47]. TUNEL+ cells in the ONL were counted throughout the full length of each retinal section along the para-sagittal plane (supero-inferior) and also included the optic nerve for consistency. These counts were subsequently averaged for each experimental group. To quantify photoreceptor survival, sections were stained with bisbenzimide (Sigma-Aldrich Co., MO, USA) and the number of rows of photoreceptor cell bodies in the ONL were counted. Five counts were made for each retinal section and then were averaged for each experimental group.

### Immunohistochemistry

Cryosections were used for immunohistochemical analysis, using antibodies against IBA1 (1:500, Wako, Osaka, Japan) and GFAP (1:500, Dako, Agilent Technologies, CA, USA) as described in our previous study, with minor modifications [41, 44]. Fluorescence in retinal sections was captured with a laser-scanning A1^+^ confocal microscope (Nikon, Tokyo, Japan). Images panels were analysed and assembled using ImageJ (NIH, MD, USA) and Photoshop CS6 software (Adobe Systems, CA, USA).

### Quantification of infiltrating macrophages

Macrophage counts were performed on sections immunolabelled with the IBA1 marker, a label for retinal microglia and macrophages. Counts of IBA1+ cells were carried out along the full length of retinal sections cut in the para-sagittal plane (supero-inferior) within the vertical meridian, and each section also included the optic nerve to ensure consistency. Counts were made of all IBA1+ cells throughout the retina, which were split into two groups: the inner retina (ILM-OPL) and the outer retina (ONL-RPE) where activated macrophages are known to aggregate during retinal degeneration [19, 20, 48].

### Polymerase chain reaction

cDNA was prepared from 1 μg of RNA of each sample using a Tetro cDNA Synthesis Kit (Bioline Reagents, London, UK). The quantitative real-time polymerase chain reactions (qPCRs) were conducted using Taqman hydrolysis probes (Table 1) and Taqman Gene Expression Master Mix (Applied Biosystems, Life Technologies, CA, USA). These were applied following the manufacturer’s instructions and run on a QuantStudio Flex 12K instrument (Applied Biosystems), at the Biomolecular Resource Facility, JCSMR, ANU. Data analysis was performed using Expression Suite v1.0.3 software (Life Technologies, CA, USA); the fold change was determined for each gene and sample using the comparative cycle threshold (*C*_t_) method (ΔΔ*C*_t_). All data was normalised to the GAPDH reference gene, which does not change in expression over the course of retinal light damage [49, 50]. ACTB was utilised as an additional reference gene in qPCRs involving the CD11b+ isolates.

**Table 1:** TaqMan hydrolysis probes used for qPCR

Gene symbol	Gene name	Catalog number	Entrez gene ID
Ccl2	Chemokine (C–C motif) ligand 2	Rn01456716_g1	24770
Ccl3	Chemokine (C–C motif) ligand 3	Rn00564660_m1	25542
Ccl4	Chemokine (C–C motif) ligand 4	Rn00587826_m1	116637
Ccl7	Chemokine (C–C motif) ligand 7	Rn01467286_m1	287561
IL-6	Interleukin 6	Rn01410330_m1	24498
IL-1B	Interleukin 1β	Rn00580432_m1	24494
TNF	Tumour necrosis factor α	Rn00562055_m1	24835
IL-12B	Interleukin 12β	Rn00575112_m1	64546
GAPDH	Glyceraldehyde-3-phosphate dehydrogenase	Rn99999916_s1	24383
ACTB	Actin, beta	Rn00667869_m1	81822

Standard PCR was performed on RNA samples purified from FACS-isolated CD11b+ macrophages, using primers specific to chemokine receptor genes (Ccr1, Ccr2, Ccr5, Cxcr3) which are detailed in our previous investigation [41]. First-strand cDNA synthesis was performed from 50 ng of RNA using the Tetro cDNA Synthesis Kit (Bioline), and standard PCR was then performed using MyTaq DNA polymerase (Bioline); negative controls had cDNA omitted from the reaction cocktail. The presence of PCR product and specificity of the reaction were assessed by gel electrophoresis.

### In situ hybridisation

Ccl3 was cloned from a 540 bp PCR product using cDNA prepared from rat retinas (as described in the qPCR section), and which was synthesised into a digoxigenin (DIG)-labelled riboprobe according to our previous publication [41]. The in situ hybridisation was performed using our established protocol [51]; the Ccl3 riboprobe was hybridised on retinal cryosections overnight at 55 °C and then washed in decreasing concentrations of saline sodium citrate (pH 7.4) at 60 °C. The bound probe was visualised with either NBT/BCIP or HNNP/Fast-Red (Roche).

### Statistical analysis

All graphing and statistical analysis was performed using Prism 6 (GraphPad Software, CA, USA). A two-way ANOVA with Tukey’s multiple comparison post-test or an unpaired Student *t* test was utilised to determine the statistical outcome; a *P* value of <0.05 was considered statistically significant.

## Results

### Stability and localisation of NR58-3.14.3 in injected retinas

**Fig. 1:**
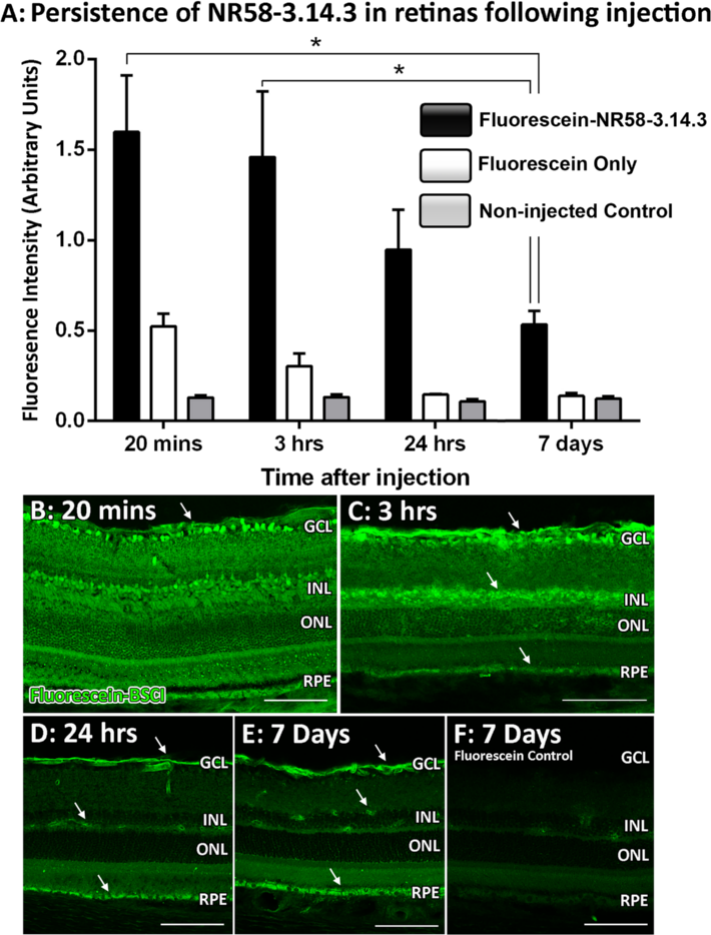
Stability and localisation of intravitreally injected NR58-3.14.3. **a** Levels of fluorescence readily increased in retinas injected with fluorescein-conjugated NR58-3.14.3 after only 20 min and remained highly elevated after 3 h. Fluorescence was significantly lower at 7 days post-injection (*P* < 0.05), but was still far higher than fluorescein-only- and non-injected controls (*P* < 0.05). **b** NR58-3.14.3-fluorescein (*green*, *arrow*) was present throughout the retina at 20 min post-injection. **c** Fluorescence remained consistent in retinas after 3 h, though was more concentrated within the GCL, NFL, INL, and RPE (*arrows*). **d**, **e** After 24 h and 7 days post-injection, fluorescence for NR58-3.14.3-fluorescein was markedly reduced but still readily apparent around the NFL, retinal vasculature, and RPE (*arrows*). **f** Retinas injected with fluorescein-only showed only very faint background fluorescence in comparison to NR58-3.14.3-fluorescein-injected animals (**e**). *GCL* ganglion cell layer, *INL* inner nuclear layer, *NFL* nerve fibre layer, *ONL* outer nuclear layer, *RPE* retinal pigment epithelium. Scale bars represent 100 μm. NR58-3.14.3-fluorescein *n* = 3, fluorescein-only *n* = 3, non-injected *n* = 3

Localisation and persistence of NR58-3.14.3 within the retina was determined by intravitreal injections of fluorescein-conjugated NR58-3.14.3 (Fig. 1). At 20 min post-injection, levels of fluorescence increased dramatically in retinas injected with NR58-3.14.3-fluorescein, compared to fluorescein-only- and non-injected controls (*P* < 0.05, Fig. 1a). Fluorescence levels were maintained at 3 h post-injection, though were reduced by approximately a third after 24 h—this was not significant however compared to the 20 min post-injection group (*P* > 0.05). After 7 days, fluorescence was still present in NR58-3.14.3-fluorescein-injected retinas at levels significantly higher than the fluorescein-only- and non-injected control groups (*P* < 0.05).

At 20 min-post-injection, NR58-3.14.3-fluorescein was distributed throughout the retina, spanning the ganglion cell layer (GCL) to the RPE (Fig. 1b). This distribution was mostly unchanged after 3 h, although fluorescence appeared more concentrated within the GCL, nerve fibre layer (NFL), inner nuclear layer (INL), and RPE (Fig. 1c, arrows). After 24 h incubation, fluorescence was localised in intense clusters encompassing the NFL, retinal vasculature, and RPE (Fig. 1d, arrows). This localisation was maintained at 7 days post-injection (Fig. 1e, arrows), while fluorescein-only controls showed little fluorescence at all (Fig. 1f).

### Tolerance of NR58-3.14.3 within the retinal environment

**Fig. 2:**
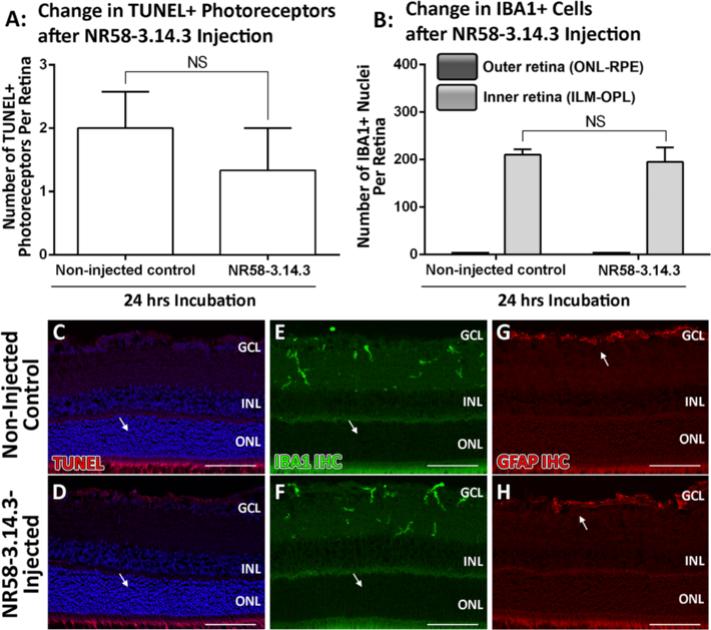
Tolerance of NR58-3.14.3 within the retinal environment in relation to stress and macrophage recruitment. **a** Injection of dim-reared controls with NR58-3.14.3 did not yield any change in TUNEL+ photoreceptors after 24 h incubation, compared to non-injected controls (*P* > 0.05). **b** NR58-3.14.3 alone did not induce any recruitment of IBA1+ macrophages to the outer retina after 24 h incubation in dim-reared animals, and there was also no change in the inner retinal population (*P* > 0.05). **c**, **d** The integrity of the ONL was unchanged in animals injected with NR58-3.14.3, compared to non-injected controls. **e**, **f** The morphology of IBA1+ macrophages/microglia (*green*) was unaltered in NR58-3.14.3-treated retinas, and the ONL was devoid of any of these cells (*arrows*) in both groups. **g**, **h** Immunoreactivity for stress marker GFAP was restricted to the GCL in both NR58-3.14.3 and non-injected groups (*arrows*), consistent with a normal physiological state. *GCL* ganglion cell layer, *IHC* immunohistochemistry, *INL* inner nuclear layer, *NFL* nerve fibre layer, *ONL* outer nuclear layer, *RPE* retinal pigment epithelium. Scale bars represent 100 μm. NR58-3.14.3 *n* = 3, non-injected *n* = 3

NR58-3.14.3 was initially injected into dim-reared retinas to determine whether it induced any change in photoreceptor death, retinal stress, or macrophages alone (Fig. 2). At 24 h following intravitreal injection with NR58-3.14.3, analyses showed no significant change in the number of TUNEL+ photoreceptors (*P* > 0.05, Fig. 2a) or in integrity of the retinal layers (Fig. 2c, d), compared to control retinas; both parameters were consistent with our previous measurements in control SD retinas [44]. General retinal stress was assessed via immunohistochemistry for GFAP (Fig. 2g, h), an accepted indicator of retinal pathology [52]. Immunoreactivity for GFAP was confined to the GCL (arrows) in both NR58-3.14.3- and PBS-injected retinas, consistent with a normal physiological retinal state [44, 52, 53]. Finally, there was no change in the distribution of IBA1+ microglia/macrophages in NR58-3.14.3-injected retinas compared to PBS-injected controls (*P* > 0.05, Fig. 2b, e, f). The outer nuclear layer (ONL) and subretinal space was devoid of macrophages in both groups (Fig. 2e, f, arrows), while resident microglia/macrophages in the inner retina showed no change in number or morphology.

### Effect of NR58-3.14.3 on macrophage accumulation following light damage

**Fig. 3:**
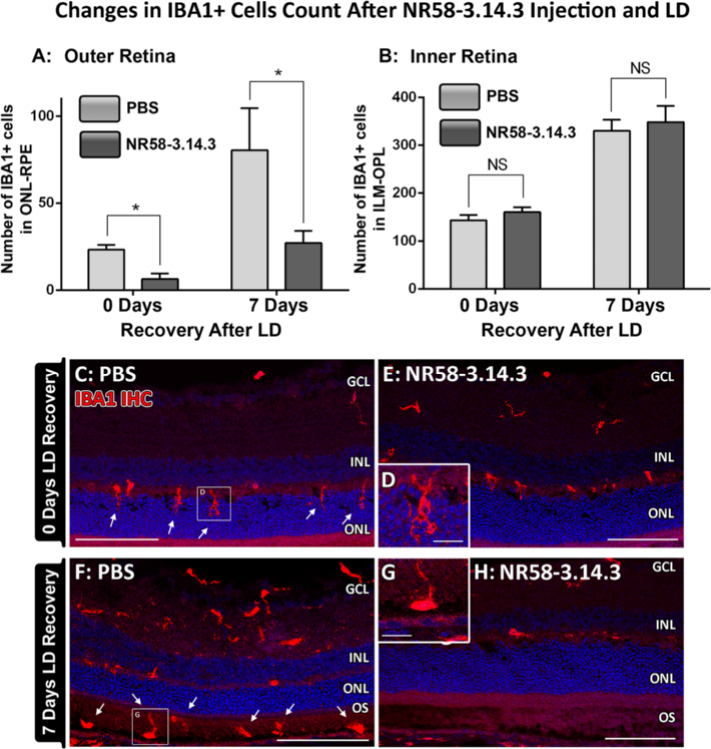
Macrophage/microglia recruitment to the outer retina following injection of NR58-3.14.3 and light damage. **a**, **b** At 0 day post-LD, there was a substantial incursion of IBA1+ cells and their ramified processes into the outer retina of PBS-injected controls, which was substantially lower in animals injected with NR58-3.14.3 (*P* < 0.05). During the chronic phase of degeneration at 7 days post-LD, the number of IBA1+ cells was further increased in the outer retina, although animals injected with NR58-3.14.3 had far fewer IBA1+ cells than in PBS controls (*P* < 0.05). For the inner retinal counts of IBA1+ macrophages/microglia, there was no change at either 0 or 7 days recovery for both treatment groups (*P* > 0.05). **c**–**e** IBA1+ cells invaded ONL with their extended processes in PBS-injected animals at 0 day (**c**, **d**, *arrows*), which was not apparent in the NR58-3.14.3 group (**e**). **f**–**h** Accumulations subretinal IBA1+ macrophages were observed in PBS-injected animals at 7 days (**f**, **g**, *arrows*), while the subretinal space was nearly devoid of these cells in NR-injected retinas. *GCL* ganglion cell layer, *IHC* immunohistochemistry, *INL* inner nuclear layer, *NFL* nerve fibre layer, *ONL* outer nuclear layer, *RPE* retinal pigment epithelium. **c**–**h** Scale bars represent 100 μm and **d**, **g** 25 μm. *N* = 11 per group at 0 and 7 days

The accumulation of macrophages within the outer retina (including the photoreceptor layer and subretinal space) following NR58-3.14.3 injection was assessed using IBA1 immunoreactivity (Fig. 3) to identify microglia/macrophages [54, 55]. Immediately following light damage (0 day recovery), we observed a substantial incursion of IBA1+ cells into the outer retina of PBS-injected controls (23 per retinal section, Fig. 3a, c, d arrows). In animals injected with NR58-3.14.3, this accumulation was substantially less at 6 per retinal section (*P* < 0.05) (Fig. 3a, e).

In PBS-injected animals 7 days post-light damage, the number of IBA1+ cells increased significantly throughout the retina, particularly in the subretinal space (Fig. 3a) where accumulations of swollen, amoeboid-like macrophages were observed among the degenerating photoreceptor segments (Fig. 3f, g arrows). In animals injected with NR58-3.14.3, however, the number of IBA1+ cells infiltrating the outer retina was significantly less than in PBS controls (27 and 80 per retina respectively, *P* < 0.05, Fig. 3a), and virtually no macrophages were detected in the subretinal space (Fig. 3h). Conversely, the numbers of IBA1+ cells in the inner retina was similar in NR58-3.14.3- and PBS-injected animals at both 0 and 7 days recovery (*P* > 0.05, Fig. 3b).

### Change in photoreceptor integrity following NR58-3.14.3 injection and light damage

**Fig. 4:**
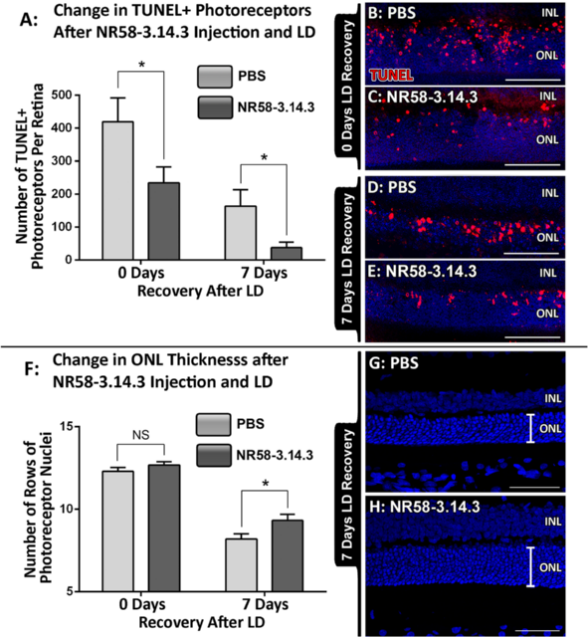
Change in photoreceptor integrity following injection of NR58-3.14.3 and light damage. **a** At 0 day post-LD, animals injected with NR58-3.14.3 had approximately half the number of TUNEL+ cells per retina compared to PBS-injected controls (*P* < 0.05). After 7 days post-LD, where chronic retinal degeneration is most apparent, the number of TUNEL+ photoreceptors was roughly three times lower in animals injected with NR58-3.14.3 than in the PBS controls (*P* < 0.05). **b**–**e** Representative TUNEL images showcase the reduction in TUNEL+ photoreceptors in NR58-3.14.3-injected retinas at both 0 (**c**) and 7 (**e**) days post-LD, compared to PBS controls (**b**, **d**). **f** At 0 day post-LD, there was no change in the number of rows of photoreceptor nuclei in NR58-3.14.3-injected retinas compared to PBS controls (*P* > 0.05). However, at 7 days post-LD, animals injected with NR58-3.14.3 had a larger number of remaining photoreceptor rows than PBS controls (*P* < 0.05). **g**, **h** Representative ONL images demonstrate this reduction of photoreceptor loss in NR58-3.14.3-injected retinas compared to PBS controls at 7 days post-LD. *INL* inner nuclear layer, *ONL* outer nuclear layer. **b**–**e** Scale bars represent 100 μm and **g**–**j** 50 μm. *N* = 10–11 per group at 0 day, *N* = 12 per group at 7 days

The effect of NR58-3.14.3 administration on photoreceptor cell death following light-induced damage was investigated using the TUNEL assay (Fig. 4). Exposure to bright continuous light induced an increase in the number of TUNEL+ photoreceptors in both PBS- and NR58-3.14.3-injected groups (Fig. 4a). However, the number of TUNEL+ cells in animals injected with NR58-3.14.3 was approximately half that in the PBS-injected control animals at 0 day recovery (235 and 419 per retinal section, *P* < 0.05, Fig. 4a–c). The effect of NR58-3.14.3 was even more pronounced during the chronic phase of degeneration at 7 days recovery, wherein the number of TUNEL+ photoreceptors was four times lower in NR58-3.14.3-injected animals compared with the PBS controls (38 and 164 per retinal section, *P* < 0.05, Fig. 4a, d, e). In addition, the cumulative effect of NR58-3.14.3 on the number of surviving photoreceptors following light damage was determined via measurements of ONL thickness. There was no change in the number of photoreceptor rows at 0 day recovery compared to PBS controls (*P* > 0.05, Fig. 4f). At 7 days recovery, however, there was an increased number of photoreceptor rows in NR58-3.14.3 retinas compared to those in PBS controls (*P* < 0.05, Fig. 4f–h).

### Cytokine and chemokine expression following NR58-3.14.3 injection and light damage

**Fig. 5:**
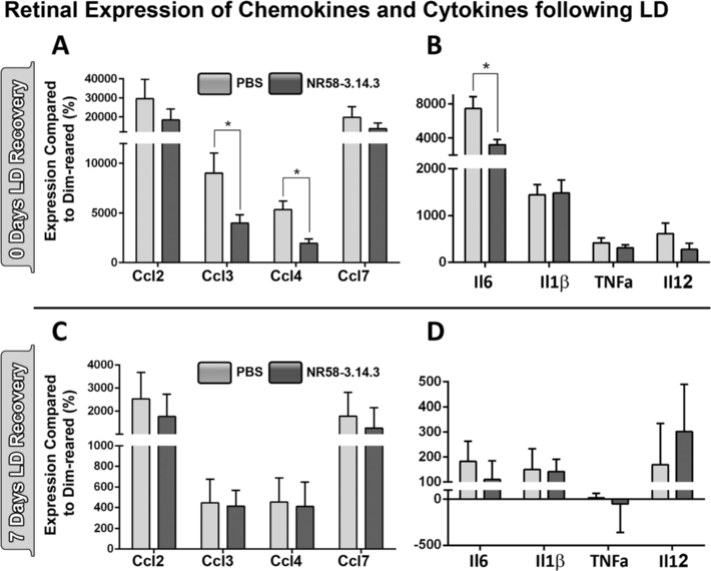
Expression of chemokines (**a**, **c**) and cytokines (**b**, **d**) in whole retinas following injection of NR58-3.14.3 and light damage. **a**, **b** All genes assessed exhibited significant up-regulation at 0 day post-LD in both treatment groups. However, Ccl3, Ccl4, and Il6 had significantly lower expression (*P* < 0.05) in retinas injected with NR58-3.14.3 compared to PBS controls. **c**, **d** By 7 days post-LD, up-regulation of all genes assessed was only 10–20 % of that observed at 0 day, with no difference between NR58-3.14.3- and PBS-injected groups (*P* > 0.05). 0 day *N* = 11–12 per group, 7 days *N* = 5 per group

The expression of chemokines (Ccl2, Ccl3, Ccl4, Ccl7) and cytokines (Il6, Il1b, TNFa, Il12) was assessed using qPCR to determine the effect of NR58-3.14.3 on chemokine signalling (Fig. 5). These were selected based on their up-regulation in our microarray analysis conducted previously using the light damage model [41]. Expression levels of all genes were substantially up-regulated immediately after bright light exposure (0 day) in both PBS- and NR58-3.14.3-injected animals (Fig. 5a, b). However, expression levels of Ccl3, Ccl4, and Il6 were significantly less in NR58-3.14.3-injected compared to PBS-injected animals (Fig. 5a, b; *P* < 0.05). At 7 days post-light damage, expression levels of all cytokines/chemokines assessed had dropped by approximately 80–90 % (Fig. 5c, d) and there was no difference between expression levels in the two treatment groups (*P* > 0.05).

### Change in expression of inflammatory factors by macrophages following NR58-3.14.3 injection and light damage

**Fig. 6:**
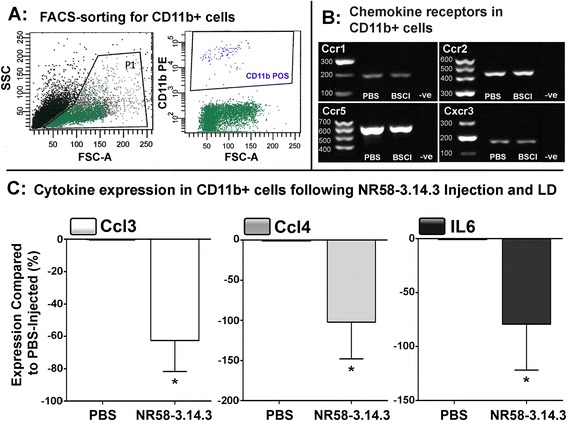
Effect of NR58-3.14.3 on the pathogenic phenotype of the retinal macrophage population following light damage. **a** Representative FACS plots, with gating strategies for the isolation of CD11b+ macrophages/microglia. Gating methodology was applied equally for all samples. **b** Representative images of PCR products via electrophoresis for chemokine receptors Ccr1, Ccr2, Ccr5, and Cxcr3, in samples of CD11b-sorted cells. Receptor expression was observed in both NR58-3.14.3 and PBS CD11b isolates. **c** Following exposure to LD, expression of Ccl3, Ccl4, and Il6 were all decreased in CD11b+ macrophages isolated from NR58-3.14.3-injected retinas, compared to those from PBS-injected retinas (*P* < 0.05). *N* = 5 per group at 0 day

Macrophages/microglia were FACS-sorted from retinal suspensions for each treatment group on the basis of CD11b immunoreactivity (Fig. 6a), which co-localises with IBA1 [56–58]. CD11b was preferred over IBA1 as a macrophage/microglial marker in this instance, because the intracellular staining required for IBA1 would prevent any downstream RNA analysis following FACS. We first sought to confirm whether the CD11b+ macrophages expressed Ccl- and Cxcl- chemokine receptors which correspond to the ligands up-regulated following light damage [41], as well as in AMD [28]. Using standard PCR (Fig. 6b), CD11b+ macrophages/microglia from both treatment groups were found to express Ccr1, Ccr3, Ccr5, and Cxcr3 receptors.

**Fig. 7:**
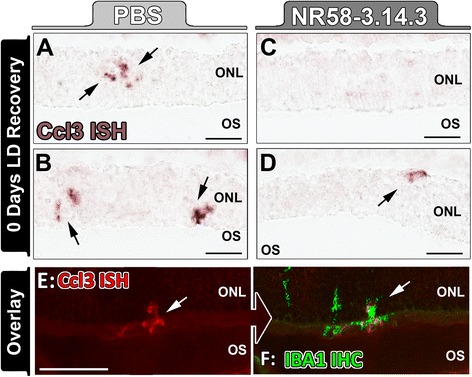
In situ hybridisation for Ccl3 mRNA following NR58-3.14.3 injections and light damage. **a**, **b** At 0 day LD recovery in PBS-injected retinas, there was staining for Ccl3 mRNA (*purple*) in among clusters of irregularly shaped nuclei traversing the ONL (*arrows*). **c**, **d** Retinas injected NR58-3.14.3, conversely, displayed very few Ccl3-expressing nuclei. **e**, **f** Ccl3-expressing nuclei (*red*, *arrow*) correlated strongly with immunofluorescence for IBA1+ macrophages/microglia (*green*, *arrow*). *IHC* immunohistochemistry, *ISH* in situ hybridisation, *ONL* outer nuclear layer, *OS* outer segments. Scale bars represent 50 μm

To further explore the role of macrophages/microglia in retinal inflammation, we compared the expression of Ccl3, Ccl4, and Il6 in isolated CD11b+ macrophages from NR58-3.14.3-injected and PBS-injected groups using qPCR. These were chosen due to their differential expression in the whole-retina samples (shown in Fig. 5) and the implicit roles of these genes in promoting pathogenic macrophage activity [34, 59]. After light damage, expression of Ccl3, Ccl4, and Il6 were significantly decreased in CD11b+ cells from NR58-3.14.3-injected retinas, compared to PBS controls (*P* < 0.05, Fig. 6c). In situ hybridisation for Ccl3 mRNA showed clusters of ramified microglia in the outer retina of PBS controls (Fig. **7**a, b arrows) following light damage, which were also immunoreactive for IBA1 (Fig. **7**e, f arrows). Ccl3-expressing macrophages were far less numerous in retinas from NR58-3.14.3-injected animals (Fig. **7**c, d arrows).

## Discussion

The findings of the current study demonstrate the efficiency of the broad-spectrum chemokine inhibitor NR58-3.14.3 in modulating the pathogenic activity of macrophages/microglia and ameliorating photoreceptor death, in a model of photo-oxidative damage and outer retinal inflammation. First, our data demonstrate that the intravitreal delivery of NR58-3.14.3 is tolerated within the retinal environment, and its persistence in the retina provides for a generous window of activity. Second, we show that injection of NR58-3.14.3 suppresses the chronic accumulation of macrophages/microglia within the photoreceptor layer and subretinal space following damage. Third, NR58-3.14.3-treated macrophages demonstrated reduced pathogenic activation, indicated by decreased expression of Ccl3, Ccl4, and Il6. Finally, we show that photoreceptor degeneration is reduced across the time course of damage in retinas injected with NR58-3.14.3, which is accompanied by an increase in photoreceptor survival at the site of focal and progressive atrophy.

NR58-3.14.3 is thought to exert its inhibitory effect on Ccl- and Cxcl- families though disruption of intracellular signal transduction, which effectively renders leukocytes blind to directional cues provided by Ccl- and Cxcl- ligands [60, 61]. This broad suppression is desirable, as the blockade of singular chemokine ligands, such as Ccl2, can induce compensatory increases in the expression other chemokines which exacerbate retinal inflammation and degeneration [34, 36]. Up-regulation of Ccl2, Cxcl1, Cxcl10, and Cxcl11 are present in all forms of AMD [28], and the Ccl2/Ccr2 axis is implicated in the pathogenic accumulation of macrophages to the outer retina in models that feature aspects of either CNV or geographic atrophy, such as light damage [16, 31–35]. Increases in ocular Ccl- and Cxcl- chemokines are also found in individuals affected by retinal detachment [62, 63], uveitis [64], glaucoma [65], diabetic retinopathy, and retinitis pigmentosa [66], while blockade of Ccl2 signalling has proven beneficial in thwarting macrophage aggregation and cell death in experimental retinal detachment, diabetic retinopathy [23], and retinitis pigmentosa [67]. While the light damage paradigm does not model features of CNV or degenerations such as retinal detachment, our findings allude to the potential of BSCIs in targeting a broad swathe of chemokine activity across a spectrum of retinal degenerations.

To our knowledge, this is the first study to examine the potential of BSCIs as a potential therapeutic agent in retinal disease. The findings from our study are consistent with previous investigations using NR58-3.14.3 in inflammatory models ranging from atherosclerotic plaques to obliterative bronchiolitis, wherein an inhibition of macrophage accumulation and amelioration of tissue damage were observed [61, 68, 69]. Our own data show that intravitreal NR58-3.14.3 reduces the accumulation of macrophages within the outer retina, including ONL and subretinal space. The proportion of these subretinal macrophages that are either resident microglia or recruited bone-marrow-derived monocytes is unclear, although the effect of intravitreal NR58-3.14.3 on these subpopulations is beyond the scope of this study. Nevertheless, we speculate that intravitreal NR58-3.14.3 could impact the infiltration of both subpopulations to the subretinal space, despite the local nature of its administration. This is suggested by the localisation fluorescein-NR58-3.14.3 within the retinal vasculature, where blood borne monocytes are known to recruit into the retina following light-induced injury [36].

Our data indicate that NR58-3.14.3 modulates the transcriptional phenotype of macrophages following light damage. The expression of Ccl3, Ccl4, and Il6 were all down-regulated in macrophages exposed to NR58-3.14.3 and are consistent with the down-regulation of Il6 observed in experimental obliterative bronchiolitis following administration of NR58-3.14.3 [39]. Ccl3, Ccl4, and Il6 are markers of M1 polarisation, which is typically associated with deleterious pro-inflammatory responses [70]. Although macrophage polarisation is considered a simplification of extremes which form part of a phenotypic spectrum, the suppression of these genes within the macrophage population in NR58-3.14.3-injected retinas do point toward a lower inflammatory state among these cells as a result. How NR58-3.14.3 elicits this transcriptional change is unclear, though we speculate it may occur via its inhibition of macrophage chemotaxis to the outer retina, wherein emergent photoreceptor death may stimulate up-regulation of cytokines such as Ccl3, Ccl4, and Il6. This is supported by our in situ hybridisation data, wherein Ccl4 mRNA is expressed only in macrophages that have migrated to the outer retina.

Several lines of evidence suggest that Ccl3, Ccl4, and Il6 are important mediators of pathogenic activity by macrophages in retinal degeneration, which may account for the neuroprotective action of NR58-3.14.3. Increased levels of Il6 are associated with the incidence and progression of AMD, and the cytokine is considered a potential therapeutic target [71–73]. A pathogenic role of Il6 is more recently highlighted by Levy and colleagues, who show that accumulation of the AMD biomarker apolipoprotein E (APOE) in subretinal macrophages up-regulates their expression of Il6, which in turn promotes their increased survival and induction of retinal pathology in light-damaged Cx3cr1−/− mice [59]. Additionally, a deleterious role of Il6 has been characterised in experimental ocular toxoplasmosis via antibody neutralisation, which considerably improved retinal morphology compared to controls [74].

Ccl3 is implicated in animal models of AMD/Stargardt disease (Abca4−/−Rdh8−/−), retinitis pigmentosa (Mertk−/−) [34], and oxygen-induced retinopathy [75]. Ccl3 was expressed by macrophages/microglia as shown by Kohno and colleagues—consistent with our current investigation—and its ablation reduced the extent of photoreceptor degeneration in both aged Abca4−/−Rdh8−/− mice and young Mertk−/− mice [34]. In experimental oxygen-induced retinopathy, mice were treated with the combination therapy of Ccl3- and Ccl2-neutralising antibodies, rather than a singular therapy. Regardless, the neovascular pathology was reduced by 30 % in these animals [75]. The precise role of Ccl4 in retinal degeneration has yet to be examined, although protein levels are increased in the aqueous humour of AMD patients [76], and its expression is up-regulated concomitantly with emergence of retinal degeneration in experimental light damage [41], diabetic retinopathy, and Abca4−/−Rdh8−/− and Mertk−/− mice [34]. Moreover, ablation of Ccl3 induced compensatory increases in Ccl4 expression in light-damaged Abca4−/−Rdh8−/− mice, which was postulated to contribute to the increased pathology observed in this paradigm (rather than the age paradigm where there was protection).

We speculate that NR58-3.14.3 may also confer retinal protection by inhibiting other macrophage processes, such as aberrant phagocytosis. This phenomenon has been recently demonstrated in the rd10 model of retinitis pigmentosa, in which infiltrating microglial cells were found to exacerbate cell death by phagocytosing non-apoptotic photoreceptors [77]. In suppressing the infiltration of macrophages within the outer retina, NR58-3.14.3 may also reduce the erratic phagocytosis of photoreceptors by activated macrophages. This may be a contributing factor to the increases in ONL thickness in NR58-3.14.3-injected retinas, although additional investigations are required to firmly establish this in the future.

## Conclusions

Our findings showcase the efficacy of BSCIs such as NR58-3.14.3 in modulating macrophage/microglia responses in retinal degeneration. NR58-3.14.3 ameliorated the chronic aggregation of subretinal macrophages in light-induced subretinal inflammation and suppressed their potentiation of Il6, Ccl3, and Ccl4 signalling. As with all current models, light damage does not encompass every aspect of AMD pathology; nevertheless, our findings have implications for the treatment of inflammation in the disease. Chemokines are associated with the aggregation of subretinal macrophages in AMD, while expression of Il6, Ccl3, and Ccl4 are implicated in augmenting their survival and pathogenicity within the subretinal space. Together, these experiments offer proof-of-principle for the potential of BSCIs as therapeutic agents in targeting subretinal inflammation in diseases such as AMD.
